# Biallelic Mutations in *NBAS* Cause Recurrent Acute Liver Failure with Onset in Infancy

**DOI:** 10.1016/j.ajhg.2015.05.009

**Published:** 2015-07-02

**Authors:** Tobias B. Haack, Christian Staufner, Marlies G. Köpke, Beate K. Straub, Stefan Kölker, Christian Thiel, Peter Freisinger, Ivo Baric, Patrick J. McKiernan, Nicola Dikow, Inga Harting, Flemming Beisse, Peter Burgard, Urania Kotzaeridou, Joachim Kühr, Urban Himbert, Robert W. Taylor, Felix Distelmaier, Jerry Vockley, Lina Ghaloul-Gonzalez, Johannes Zschocke, Laura S. Kremer, Elisabeth Graf, Thomas Schwarzmayr, Daniel M. Bader, Julien Gagneur, Thomas Wieland, Caterina Terrile, Tim M. Strom, Thomas Meitinger, Georg F. Hoffmann, Holger Prokisch

**Affiliations:** 1 Institute of Human Genetics, Technische Universität München, 81675 München, Germany; 2 Institute of Human Genetics, Helmholtz Zentrum München, 85764 Neuherberg, Germany; 3 Department of General Pediatrics, Division of Neuropediatrics and Metabolic Medicine, University Hospital Heidelberg, 69120 Heidelberg, Germany; 4 Institute of Pathology, University Hospital Heidelberg, 69120 Heidelberg, Germany; 5 Children’s Hospital Reutlingen, 72764 Reutlingen, Germany; 6 Department of Pediatrics, University Hospital Center Zagreb and University of Zagreb, School of Medicine, 10000 Zagreb, Croatia; 7 Liver Unit, Birmingham Children’s Hospital, Birmingham B4 6NH, UK; 8 Institute of Human Genetics, University Hospital Heidelberg, 69120 Heidelberg, Germany; 9 Department of Neuroradiology, University Hospital Heidelberg, 69120 Heidelberg, Germany; 10 Ophthalmology Department, University Hospital Heidelberg, 69120 Heidelberg, Germany; 11 Children’s Hospital Karlsruhe, 76133 Karlsruhe, Germany; 12 Children’s Hospital St. Elisabeth, 56564 Neuwied, Germany; 13 Wellcome Trust Centre for Mitochondrial Research, Institute of Neuroscience, The Medical School, Newcastle University, Newcastle upon Tyne NE2 4HH, UK; 14 Department of General Pediatrics, Neonatology and Pediatric Cardiology, University Children’s Hospital, Heinrich-Heine-University Düsseldorf, 40225 Düsseldorf, Germany; 15 University of Pittsburgh School of Medicine, Children’s Hospital of Pittsburgh of UPMC, Pittsburgh, PA 15224, USA; 16 Division of Human Genetics, Innsbruck Medical University, 6020 Innsbruck, Austria; 17 Computational Genomics, Gene Center, Ludwig Maximilians University, 81377 Munich, Germany

## Abstract

Acute liver failure (ALF) in infancy and childhood is a life-threatening emergency. Few conditions are known to cause recurrent acute liver failure (RALF), and in about 50% of cases, the underlying molecular cause remains unresolved. Exome sequencing in five unrelated individuals with fever-dependent RALF revealed biallelic mutations in *NBAS.* Subsequent Sanger sequencing of *NBAS* in 15 additional unrelated individuals with RALF or ALF identified compound heterozygous mutations in an additional six individuals from five families. Immunoblot analysis of mutant fibroblasts showed reduced protein levels of NBAS and its proposed interaction partner p31, both involved in retrograde transport between endoplasmic reticulum and Golgi. We recommend *NBAS* analysis in individuals with acute infantile liver failure, especially if triggered by fever.

## Main Text

Acute liver failure (ALF) is a very severe, life-threatening event which can result from toxin exposure, inherited metabolic disease, autoimmune disease, infectious disease, shock, and other rare causes. The exact incidence of pediatric ALF is unknown, and in about 50% of cases the underlying cause remains obscure.^1,2^ Prognosis is guarded and emergency liver transplantation is often the only therapeutic option. Known causes of recurrent acute liver failure (RALF) with clinical and biochemical hepatic recovery in the interval include fulminant viral hepatitis, autoimmune hepatitis, disorders of long-chain fatty acid oxidation and the carnitine cycle, dihydrolipoamide dehydrogenase (E3) deficiency (MIM: 246900), and Wolcott-Rallison syndrome (MIM: 226980).^3–7^ Here we report the identification of homozygous or compound heterozygous mutations in *NBAS* (neuroblastoma amplified sequence) in 11 individuals with RALF starting in infancy.

**Figure 1:**
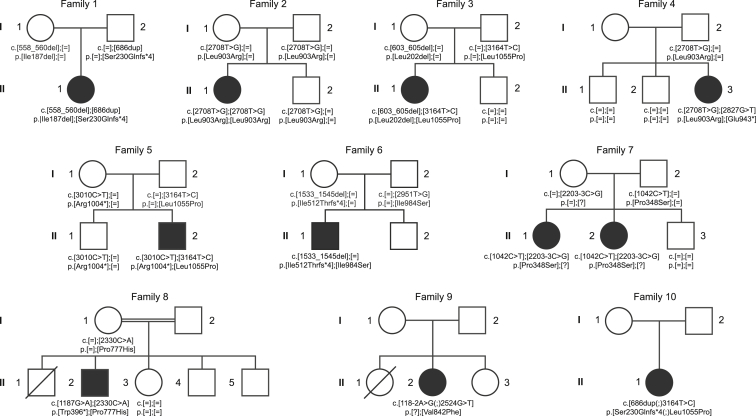
Pedigrees of Investigated Families Pedigrees of ten families affected by RALF and mutations in *NBAS*. Mutation status of affected (closed symbols) and healthy (open symbols) family members.

**Figure 2:**
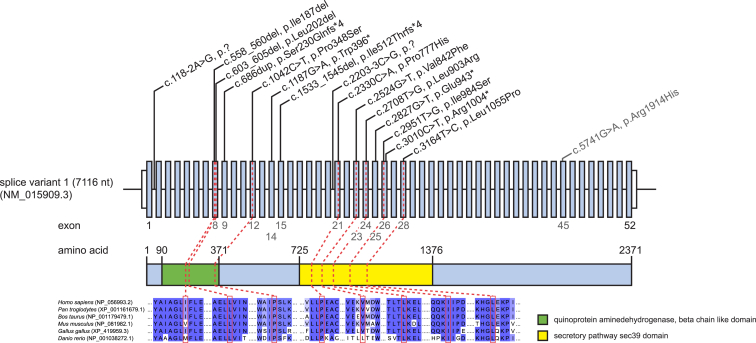
*NBAS* Structure and Conservation of Identified Mutations Gene structure of *NBAS* with known protein domains of the gene product and localization and conservation of amino acid residues affected by mutations. Mutation c.5741G>A (p.Arg1914His) in exon 45 in the C-terminal domain of unknown function is associated with SOPH syndrome.^17^ Amino acids 90–371 form a quinoprotein aminedehydrogenase, beta chain like domain (IPR011044) and amino acids 725–1,376 form a secretory pathway sec39 domain (IPR013244). Intronic regions are not drawn to scale.

A group of four unrelated German individuals with previously unclassified early-onset RALF (according to the definition of the Pediatric Acute Liver Failure Study Group)^1^ was recruited in one clinical center (University Hospital Heidelberg) and jointly analyzed. Clinical and genetic findings are summarized in Table 1. Informed consent to participate in the study was obtained from all affected individuals (or their parents, in the case of minor study participants). The study was approved by the ethical committees of the University Hospital Heidelberg and of the Technische Universität München. Whole exome sequencing was performed on genomic DNA from four unrelated affected individuals as described previously.^8^ Using a frequency filter of minor allele frequency < 0.1% in our in-house database and public databases, we identified six, seven, three, and nine compound heterozygous or homozygous variants in individuals F1:II.1, F2:II.1, F3:II.1, and F4:II.3, respectively. *NBAS* (GenBank: NM_015909.3, MIM: 608025) is the only gene where biallelic mutations were identified in all affected individuals (Figure 1). In individual F5:II.2, presumably disease-causing mutations in *NBAS* had been identified in a preceding exome sequence analysis but the relevance of this finding had remained unclear. The analysis of exome sequencing data from approximately 4,000 individuals with unrelated phenotypes (all subjects were sequenced in the Institute of Human Genetics, Helmholtz Zentrum München, and their data stored in an in-house database) identified only one additional sample with two rare variants in *NBAS* potentially compatible with a recessive disease. However, parental samples were not available and a compound heterozygous state of the two variants has not been confirmed. We subsequently studied 15 additional unrelated individuals with unresolved RALF and ALF from four additional clinical centers by Sanger sequencing. Primer sequences and PCR conditions for Sanger sequencing of *NBAS* are available on request. This resulted in the identification of six additional individuals from five families with compound heterozygous mutations in *NBAS* (Figure 1). All 11 individuals carry at least one missense mutation on one allele. All missense mutations change evolutionarily conserved amino acid residues (Figure 2) and are accordingly predicted to be damaging (PolyPhen-2 and SIFT). Carrier testing showed that each parent analyzed was heterozygous for one allele; the mother of individual F4:II.3 had died before the study was performed so testing was impossible and parental samples of individuals F8:II.2, F9:II.2, and F10:II.1 were not available. Remarkably, in two families, F8 and F9, the oldest siblings had died in early infancy due to acute liver failure, aged 14 and 11 months, respectively; however, no material was available to genetically confirm their clinical diagnosis of NBAS deficiency.

Notably, all mutations, which are not predicted to cause a loss of function, are clustered in two regions in the first half of the gene, exons 8–12 and exons 21–28. The mutations in the latter region affect the secretory pathway sec39 domain of NBAS (Figure 2). Individual F2:II.1 carries a homozygous missense variant and individual F3:II.1 an in-frame deletion of one amino acid compound heterozygous with a missense mutation. All other affected individuals are compound heterozygotes for one out of seven different predicted loss-of-function mutations (three stop mutations, two frameshift, and two splice site mutations) plus a missense mutation or a deletion of a single amino acid. None of the *NBAS* missense mutations is present in >120,000 alleles from the Exome Aggregation Consortium (ExAC) Server (12/2014). Three of the loss-of-function mutations, c.686dup (p.Ser230Glnfs^∗^4), c.2827G>T (p.Glu943^∗^), and c.3010C>T (p.Arg1004^∗^), are listed in the heterozygous state 59×, 8×, and 1×, respectively (MAF < 0.04%). Although we have no indication for a shared haplotype for the c.3164T>C variant identified heterozygous in three families, the exome data suggests a common founder c.2708T>G mutation in German families 2 and 4.

**Figure 3:**
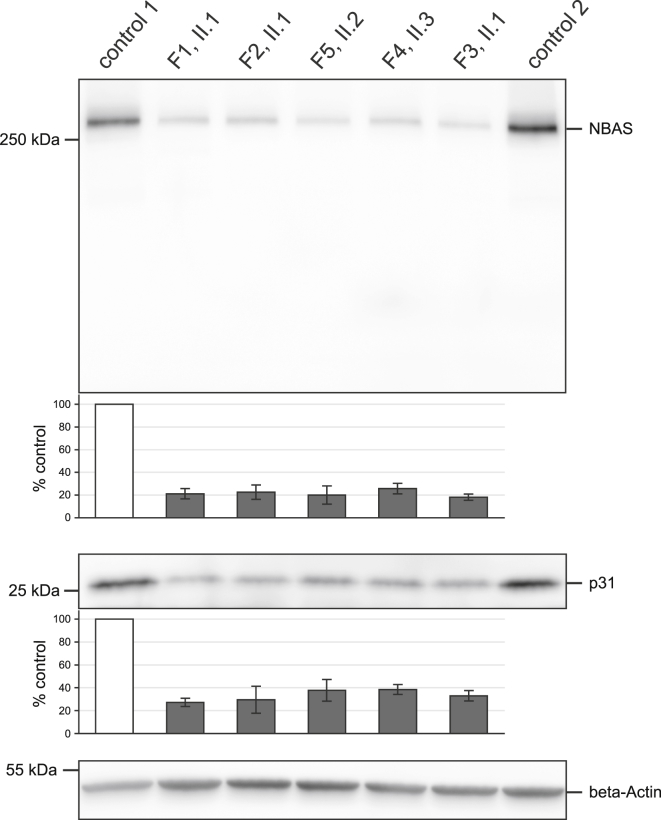
Quantification of NBAS and p31 Protein Levels Mutant and control fibroblast cell lines were cultivated at 37°C. 10 μg protein of collected cells were separated on a 4%–12% acrylamide gradient gel, transferred to a PVDF membrane, and immunodecorated with antibodies against NBAS, p31, and β-actin. The antibody for NBAS was detecting a single protein band at approximately 270 kDa corresponding to the predicted molecular weight of NBAS. β-actin was used as a loading control. The quantified protein levels are based on three independent experiments and expressed as percentages of a control cell line, corrected for β-actin. Five of the eight investigated subject cell lines are shown. NBAS and p31 protein levels were severely reduced in fibroblast cell lines of RALF-affected individuals to 21% and 33% of control subjects, respectively. Error bar indicates 1 SD. Protein amount in control 1 was set as 100%.

Next, the functional consequences of *NBAS* mutations on the protein level were investigated. In eukaryotic cells, soluble N-ethylmaleimide-sensitive factor attachment protein receptor (SNARE) tethering factors mediate the docking and fusion of transport vesicles with target membranes. Fusion of membranes is mediated by membrane-bound proteins on the transport vesicles (v-SNARE) and target membrane (t-SNARE). NBAS is thought to function as a component of an ER tethering complex that interacts with the t-SNAREs p31, BNIP1, and STX18 at the ER and the v-SNARE Sec22b. This tethering complex also includes ZW10, RINT1, and Sly1 (see Figure S1).^9,10^ Mutant and control fibroblasts were cultivated in D-MEM media supplemented with 10% FBS, 1% penicillin-streptomycin, and 200 μM uridine at 37°C and 5% CO_2_. For immunoblots, cells were collected, washed in PBS, and resolved in RIPA buffer.^11^ 10 μg of protein of every sample were separated on a 4%–12% acrylamide gradient gel (LONZA). Primary antibodies (all Sigma-Aldrich) against NBAS (1:2,000), p31 (USE-1) (1:250), and β-actin (1:15,000) were incubated overnight. Enhanced chemiluminescence of proteins was detected with a Vilberscan Fusion FX7. Protein levels were quantified with the software Bio-1D. Testing of individual F1:II.1 fibroblasts showed a significant decrease in NBAS steady-state levels (p = 0.03; two-tailed unpaired t test). Extending the quantification of NBAS protein to affected individuals from families 2–5 and 7 showed a reduction of NBAS levels to 18%–36% in comparison to controls, indicating a substantial impairment of protein translation and/or stability in all seven affected individuals investigated (Figure 3 shows representative findings for individuals from families 1–5). Moreover, the reduction of NBAS was concomitant with a reduction of its proposed interaction partner p31, supporting an important function of NBAS within the syntaxin 18 complex (Figure S1). None of the additional components have been associated with a human disorder. Mouse models of deleted p31 and RINT1 were shown to be embryonic lethal.^12,13^

Next, we investigated whether an assumed defect in the assembly of the ER tethering complex impairs glycosylation and/or results in increased ER stress. Isoelectric focusing of transferrin, alpha-1-antitrypsin, and apolipoprotein CIII followed by in-gel immunodetection was performed in sera of a control subject, a COG6-CDG individual, and five individuals with *NBAS* mutations. Although the observed patterns indicated a clear deficiency in N-/O-glycosylation in the CDG individual, no differences were observed for the *NBAS-*mutant individuals compared to controls (Figure S2). These findings suggest that under the investigated conditions NBAS deficiency does not disturb retrograde transport between ER and Golgi to the extent that obvious changes in glycosylation patterns can be observed.

**Figure 4:**
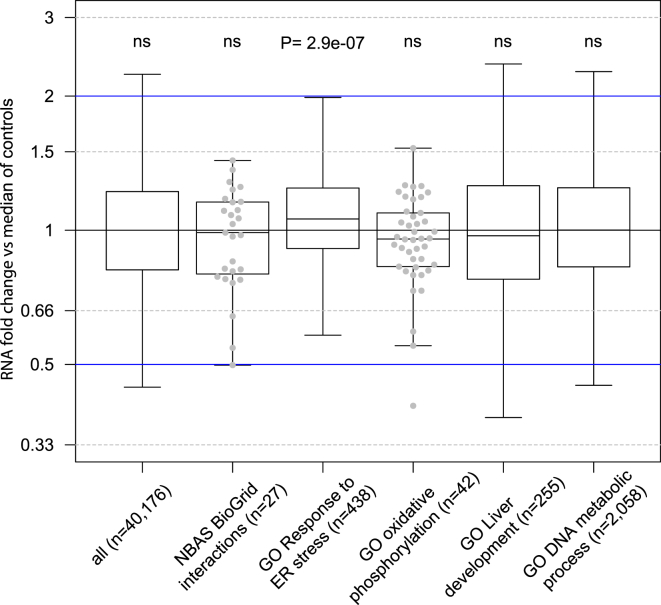
Increased Expression of ER Stress Response Genes Quartiles (boxes) and 1.5 times the interquartile range (whiskers) of the fold change of normalized RNA-seq read counts from fibroblasts are displayed. Three individuals with pathogenic variants in *NBAS* (F1:II.1, F2:II.1, and F5:II.2) are compared against the median per gene over 12 control samples. We obtained interaction partners of the NBAS protein from the BioGrid database (v.3.3).^18^ The genes associated with the corresponding gene ontology terms were downloaded from UniProt (accessed March 2015).^19^ For every box, we computed a two-sided Wilcoxon test whether it is symmetric about 1. Single data points are shown for boxes with less than 50 genes. Gene ontology (GO) terms: GO 0034976, response to endoplasmic reticulum stress; GO 0001889, liver development; GO 0006259, DNA metabolic process; GO 0006119, oxidative phosphorylation. ns, p > 0.1.

We next performed expression profiling in *NBAS-*mutant fibroblast to test for alterations suggestive for ER stress. Total cellular RNA was isolated from fibroblasts of three NBAS-affected individuals (F1:II.1, F2:II.1, F5:II.2) and 12 healthy controls. The quality of the RNA isolated from whole-cell lysates was determined with the Agilent 2100 BioAnalyzer (RNA 6000 Nano Kit, Agilent). All samples had a RNA integrity number (RIN) value greater than 8. RNA libraries were sequenced as 100 bp paired-end runs on an Illumina HiSeq2500 platform as described.^14^ In order to get expression measurements, we counted reads with HTSeq-count using the intersection-strict option on 20,345 protein coding genes from the Gencode database (GRCh37, p13, 19.07.2013).^15^ Read counts were normalized with size factors computed by DESeq2 to account for differences in sequencing depth per sample.^16^ We discarded genes with an average normalized expression below 10 over all samples and performed all consecutive analysis on the remaining 13,392 genes. Although there was no global change in gene expression, we did observe a significant increase in the expression of genes involved in ER stress response in *NBAS-*mutant fibroblasts compared to controls (Figure 4).

Together, our genetic and experimental findings provide evidence that mutations affecting functionally conserved domains in NBAS resulting in severely decreased NBAS levels cause RALF in infancy. Although no changes in glycosylation patterns were observed in the conditions investigated, expression profiles in *NBAS* mutant fibroblasts are suggestive for ER stress. RALF due to mutated *NBAS* is a presumably relatively frequent inherited cause of RALF, because *NBAS* screening of 15 individuals with RALF or acute infantile liver failure resulted in the discovery of six affected individuals from five additional families. The pivotal feature of this disease is RALF precipitated by intercurrent febrile illnesses during infancy and childhood. Intriguingly, with conservative management, liver function recovered completely and was normal in the interval. Crises are heralded by vomiting and lethargy and start rather uniformly with massively elevated ASAT and ALAT (range of maximum ALAT 4,001–17,700 U/l; normal < 50 U/l), followed by coagulopathy requiring FFP substitution (maximum INR 10; normal < 1.2) and mild to moderate jaundice (range of maximum total bilirubin 46.8–207.4 μmol/l; normal < 20.4 μmol/l). In some cases, significant hypoglycemia, hyperammonemia (maximum 209 μmol/l; normal < 53 μmol/l), and hepatic encephalopathy developed, which we consider to be secondary to ALF. Several affected individuals suffered from a range of comorbidities including cardiomyopathy, autoimmune gastrointestinal diseases, and neurological phenotypes such as epilepsy. However, none of those conditions is present in two individuals (Table 1).

Mutations in *NBAS* have not been linked to liver disease before, but a homozygous missense mutation c.5741G>A (p.Arg1914His) in the C-terminal domain of unknown function of *NBAS* has been associated with a syndrome of short stature, cone and optic nerve atrophy, and Pelger-Huët anomaly in Yakuts (SOPH syndrome [MIM: 614800]).^17^ RALF-causing NBAS deficiency and SOPH syndrome are clearly different clinical entities. Importantly, individuals with SOPH syndrome were not reported to have ALF or any hepatic disease manifestation and the impact of the SOPH-associated mutation on NBAS protein function is unknown.

None of the individuals with NBAS-induced (R)ALF carried two loss-of-function mutations in *NBAS.* However, *NBAS* mutations led to low levels of the gene product of about 25% NBAS protein (compared to healthy probands). The affected individuals recovered completely in the intervals between the crises and otherwise presented a rather mild phenotype, so reduced NBAS protein levels due to the mutations appear to be sufficient for normal function of most tissues including liver under afebrile conditions.

The physiologic function of NBAS is not yet understood. In yeast, sec39 is involved in retrograde transport between ER and Golgi.^9^ Aoki et al.^9^ provided evidence for a similar function of NBAS in humans where it is interacting with p31. Together with other SNARE proteins, they are forming the syntaxin 18 complex implicated in ER membrane fusion. Concomitant with reduced NBAS levels, p31 levels were also decreased, providing additional evidence that both proteins are subunits of the same SNARE complex. Similar findings were observed after targeted knockdown in HeLa cells.^9^ The exact mechanism of ALF due to *NBAS* deficiency remains unclear. We reason that a catabolic state with high energy demand during febrile infections or the raised temperature itself might be the starting points of the derailment, speculatively via a thermal susceptibility of the syntaxin 18 complex.

In contrast to acute infantile liver failure resulting from mutations in *TRMU* (MIM: 613070), *MARS* (MIM: 615486), or *LARS* (MIM: 615438), NBAS deficiency was not associated with increased serum lactate or muscular hypotonia. In about 50% of cases of acute infantile liver failure, the underlying cause remains undetermined. Some of these children might have an underlying genetic disorder or predisposition. The detection rate of *NBAS* mutations in random individuals with (recurrent) acute infantile liver failure was very high (6 out of 15 screened individuals), so NBAS deficiency might be a frequent cause of not only RALF but also isolated ALF at least in children. The differing genetic origins of affected individuals and variety of mutations suggest that this autosomal-recessive disease is not a regional phenomenon.

In conclusion, mutations in *NBAS* are delineated as a previously unknown cause of acute liver failure with onset in childhood triggered by febrile infections. It is possible that the affected individuals reported in this study present the severe end of the phenotypic spectrum. Notably, in families F8 and F9, two older siblings had died from acute liver failure, aged 14 and 11 months. Unfortunately, no material was available for diagnostics from the deceased individuals. However, the finding of *NBAS* mutations in their siblings suggests NBAS-induced ALF. Hence, these individuals might provide examples for a lethal course of NBAS deficiency. We recommended sequencing *NBAS* in cases of ALF in infancy and childhood of unknown cause, especially in individuals with fever-associated ALF and/or RALF.

## Figures and Tables

**Table 1:** Genetic and Clinical Findings in Individuals with *NBAS* Mutations

**ID**	**Sex**	***NBAS* Mutations**	**Protein Levels Fibroblasts**		**Clinical Features**				
**cDNA (NM_015909.3), Protein (NP_056993.2)**	**NBAS**	**p31**	**AO**	**Age Last Crisis**	**Number of ALF**	**Age Last Visit**	**Other Clinical Findings**
F1:II.1	F	c.[558_560del];[686dup], p.[Ile187del];[Ser230Glnfs^∗^4]	21%	27%	21	14 9/12 years	2	18 years	none reported
F2:II.1	F	c.[2708T>G];[2708T>G], p.[Leu903Arg];[Leu903Arg]	22%	30%	7	21 1/12 years	7	22 years	acute renal failure, epilepsy
F3:II.1	F	c.[603_605del];[3164T>C], p.[Leu202del];[Leu1055Pro]	20%	38%	10	11 7/12 years	5	18 years	celiac disease
F4:II.3	F	c.[2708T>G];[2827G>T], p.[Leu903Arg];[Glu943^∗^]	26%	38%	8	ND	ND	37 years	none reported
F5:II.2	M	c.[3010C>T];[3164T>C], p.[Arg1004^∗^];[Leu1055Pro];	18%	33%	7	9 10/12 years	10	14 years	cardiomyopathy
F6:II.1	M	c.1533_1545del];[2951T>G], p.[Ile512Thrfs^∗^4];[Ile984Ser]	ND	ND	18	3 11/12 years	5	3 years	none reported
F7:II.1	F	c.[1042C>T];[2203−3C>G], p.[Pro348Ser];[?]	32%	ND	11	6 7/12 years	4	9 years	none reported
F7:II.2	F	c.[1042C>T];[2203−3C>G], p.[Pro348Ser];[?]	36%	ND	6 8/12 years	6 8/12 years	1	11 years	erythema nodosum, Crohn’s disease
F8:II.2	M	c.[1187G>A];[2330C>A], p.[Trp396^∗^];[Pro777His]	ND	ND	21	3 years	1	8 years	none reported
F9:II.2	F	c.[118−2A>G];[2524G>T], p.[?(;)Val842Phe]	ND	ND	18	2 2/12 years	3	4 years	none reported
F10:II.1	F	c.[686dup];[3164T>C], p.[Ser230Glnfs^∗^4];[Leu1055Pro]	ND	ND	4	5 10/12 years	5	18 years	ND

NBAS and p31 protein levels are normalized to β-actin and are given in percent of a control cell line. Abbreviations are as follows: AO, age of onset (in months if not stated otherwise); M, male; F, female; ND, not determined.

## Web Resources

The URLs for the data presented herein are as follows:

ExAC Browser, http://exac.broadinstitute.org/
MutationTaster, http://www.mutationtaster.org/
OMIM, http://www.omim.org/

## Supplemental Data

Document S1. Figures S1 and S2

Document S2. Article plus Supplemental Data
