# Supplementary material for: Biallelic Mutations in NBAS Cause Recurrent Acute Liver Failure with Onset in Infancy
Source: Am J Hum Genet. 2015 Jul 2;97(1):163–9. doi: 10.1016/j.ajhg.2015.05.009 (PMC4572578; doi:10.1016/j.ajhg.2015.05.009)
Supplement: Document S1. Figures S1 and S2 [file mmc1.pdf]

The American Journal of Human Genetics
Supplemental Data

Biallelic Mutations in *NBAS* Cause Recurrent Acute Liver Failure with Onset in Infancy

Tobias B. Haack, Christian Staufner, Marlies G. Köpke, Beate K. Straub, Stefan Kölker, Christian Thiel, Peter Freisinger, Ivo Baric, Patrick J. McKiernan, Nicola Dikow, Inga Harting, Flemming Beisse, Peter Burgard, Urania Kotzaeridou, Joachim Kühr, Urban Himbert, Robert W. Taylor, Felix Distelmaier, Jerry Vockley, Lina Ghaloul-Gonzalez, Johannes Zschocke, Laura S. Kremer, Elisabeth Graf, Thomas Schwarzmayr, Daniel M. Bader, Julien Gagneur, Thomas Wieland, Caterina Terrile, Tim M. Strom, Thomas Meitinger, Georg F. Hoffmann, Holger Prokisch

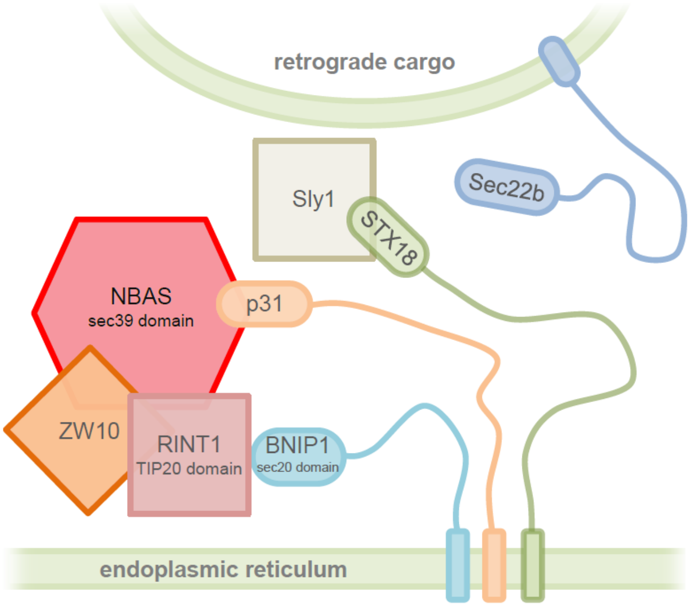

Figure S1. Proposed Function of NBAS
Schematic representation of the predicted NBAS function in the t-SNARE Syntaxin 18 complex.

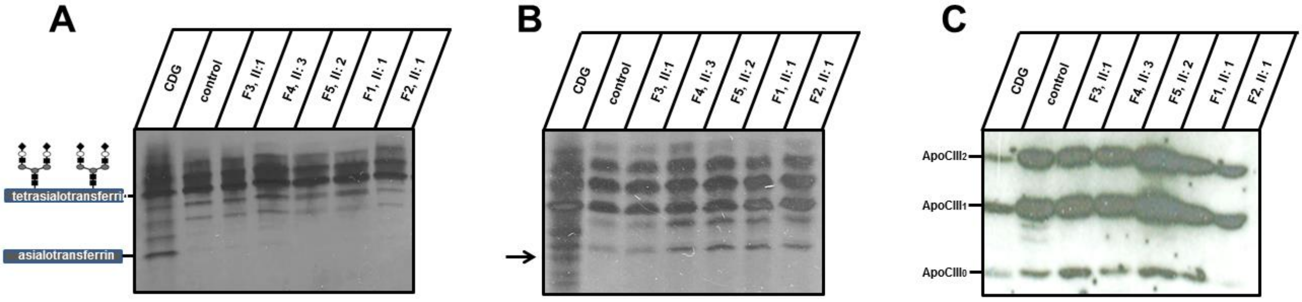

Figure S2. Isoelectric Focusing of Transferrin, Alpha-1-antitrypsin, and Apolipoprotein CIII
Sera of a control, a CDG patient and the individuals with *NBAS* variants were analyzed by isoelectric focusing (IEF), followed by in gel immunodetection.
(A) `Tetrasialo' and `asialo' indicate transferrin forms with four or no sialic acid residues on transferrin. A control-like IEF pattern was obtained for all individuals with *NBAS* variants. Filled square, N-acetylglucosamine; grey circle, mannose; open circle, galactose; filled diamond, sialic acid.
(B)For alpha-1-antitrypsin the position of the first additional abnormal cathodal band is indicated by an 'arrow'. This band and all bands below are abnormal and indicate a N-glycosylation deficiency in case of the CDG patient only.
(C) To further investigate, whether the variants in *NBAS* have an impact on the biosynthesis of core 1 mucin type O-glycans, IEF of Apolipoprotein CIII (ApoCIII) was performed. $ApoCIII_2$, $ApoCIII_1$ and $ApoCIII_0$ indicate the variability in the amount of sialic acid residues linked to ApoCIII. As for the N-glycosylation, no abnormalities were decteted in case of the individuals with *NBAS* variants.
